# Radio, Podcasts, and Music Streaming—An Electroencephalography and Physiological Analysis of Listeners’ Attitude, Attention, Memory, and Engagement

**DOI:** 10.3390/brainsci14040330

**Published:** 2024-03-29

**Authors:** Shannon Bosshard, Emma Rodero, Isabel Rodríguez-de-Dios, Jamie Brickner

**Affiliations:** 1 ARN Neurolab, Australian Radio Network, Sydney, NSW 2113, Australia; 2 Media Psychology Lab, Department of Communication, Pompeu Fabra University and UPF-Barcelona School of Management, 08002 Barcelona, Spain; 3 Department of Sociology and Communication, University of Salamanca, 37008 Salamanca, Spain; 4 Whiting School of Engineering, Johns Hopkins University, Baltimore, MD 21218, USA

**Keywords:** neuroscience, EEG, media, radio, podcasts, audio, marketing

## Abstract

Whilst radio, podcasts, and music streaming are considered unique audio formats that offer brands different opportunities, limited research has explored this notion. This current study analyses how the brain responds to these formats and suggests that they offer different branding opportunities. Participants’ engagement, attitude, attention, memory, and physiological arousal were measured while each audio format was consumed. The results revealed that music streaming elicited more positive attitudes, higher attention, greater levels of memory encoding, and increased physiological arousal compared to either radio or podcasts. This study emphasises the importance for brands of utilising diverse audio channels for unique branding and marketing opportunities.

## 1. Introduction

Audio has become ingrained in the daily lives of much of the population. In the last year, different formats have begun to complement the radio and music streaming offering [1]. According to Edison Research and Triton Digital, 68% of people listened to online audio last month, and music streaming will see its respective markets increase from approximately USD 26 billion to USD 46 billion by 2027 [2,3]. Not only has the music streaming sector seen significant growth, but podcasts have grown dramatically, with 57% of the population claiming to have listened to a podcast. The current podcast market is worth approximately USD 9.8 billion and is expected to grow at a compounded annual rate of 27.5% until 2027 [4]. Despite the past few years having been plagued with the negative effects of the pandemic, all forms of audio have seen considerable growth, including radio, which remains the most listened to audio format [5].

The subsequent growth in these channels is predominantly the result of loyal and receptive audiences, and these audiences appear to exist despite the inclusion of ads across the majority of audio formats. With regards to radio, Edison Research and National Public Radio (NPR) reported that 47% of radio listeners agreed that listening to advertisements was a fair exchange for accessing free content [6]. Similarly, the inclusion of advertising within the podcast space has not deterred audiences, given their continued inclusion and the exponentially increasing profits [7]. Specifically, 49% of podcast listeners agreed that advertising within a podcast is the best way for a brand to reach the consumer [8]. This data not only demonstrates the unique relationship that consumers have towards radio, but also demonstrates the relevance and acceptance of audio advertising. Audio is reported to be more important for individuals’ sense of themselves than what they watch or read [9]. This modality is arguably considered the most potent medium due to its ability to build consumer identity [10], create moods [11], elicit emotions [12], capture attention [13,14], target groups [15], create memories [16], change attitudes [17], and change behaviour [18,19]. For all these reasons, large organisations invest millions of dollars into the audio space to cement themselves in their consumers’ minds.

However, despite their impact and the commercial opportunities that each medium presents, research within this area is incredibly limited. The audio domain is frequently treated as a whole, even though it is generally assumed that the different formats could lead to perceptual and processing differences on the listeners’ part. At a broad level, it is clear that the audio experience differs depending on whether the format is radio, a podcast, or a music stream. While radio messages are fleeting and the listener cannot choose topics and programmes, podcasts are recorded files that can be listened to anywhere, anytime, and the listener can select the content. Therefore, podcasts lend themselves to containing more personalized or relevant content [20]. Furthermore, whilst both formats are based on the spoken word, music streaming offers an entirely different experience. However, although there are different exposure experiences and personal and motivational outcomes elicited as a result of listening to the different formats, no studies have examined the possible variations in the listeners’ perception and processing. Therefore, this study aims to analyse the listeners’ engagement, attitude, attention, and memory when exposed to the three different formats: radio, podcasts, and music streaming.

The contribution of this research is twofold. Firstly, this research aims to reveal the mechanisms that underlie the listeners’ information processing of these different audio formats. Whilst consumers of audio purport using each medium for different reasons, it is well established that conscious responses are ‘cognitively polluted’, making them more unreliable than implicit or non-conscious measures [21]. The implementation of a multidimensional approach, inclusive of neuroimaging, allows for a deeper insight into how consumption behaviour differs. Whilst surveys are a useful tool to understand top-level insights regarding consumption habits (i.e., whether participants engage in an activity), they fall short in allowing for more granular insights (i.e., why consumers engage in certain activities). Previous studies that compared the insights derived from self-report and neuroimaging have clearly shown differences in their sensitivity [17,22]. It is therefore important to understand whether each audio format is processed differently within the brain. The neurological aspects of audio consumption are fundamental, as most behaviour is driven by nonconscious processes [23].

Secondly, this paper aims to emphasise the commercial benefits of adopting a broad audio strategy encompassing numerous audio channels, should one exist. From a commercial point of view, this study aims to help audio companies and practitioners to choose the most adequate medium with regards to specific advertising strategies. If these formats are processed differently and change individuals’ personal and motivational states, then advertising within each medium could potentially offer unique marketing and advertising opportunities.

With respect to consumer behaviour, it is generally acknowledged that consumers move throughout a funnel before making a purchase. Arguably, the most commonly cited model used to describe the consumer journey is the AIDA (Awareness, Interest, Desire, and Action) model, which has been a key tool used to understand consumer behaviour for over 100 years. Therefore, it should come as no surprise that brands are most interested in how their advertising drives consumers to movement through the funnel. Neuroimaging techniques provide a direct assessment of how capable advertising is in driving changes in the perception of brands. Specifically, electroencephalography (EEG) metrics, including engagement, attitude, memory, attention, and arousal, each inform how consumers are responding to advertising and whether these responses are likely to result in behavioural change.

For the purpose of the current paper, engagement is defined as the level of immersion and presence experienced by a consumer in response to a stimulus (in this case, an ad). Engagement is of considerable interest, as it is thought to be one of the leading predictors of persuasion and, thus, advertising effectiveness [24]. Given the emphasis that brands place on understanding lower-funnel outcomes, inclusive of consideration and purchase intent, and their apparent link with engagement, the neural underpinnings of immersion/engagement are of significant relevance to the current paper. According to Kakkos et al., beta band oscillations are associated with elevated mental workload levels, a byproduct of increased immersion [25]. These findings extend upon those presented by Walter et al., who suggested that beta effects relating to increased workloads were evident across parieto-occipital electrodes [26]. Additionally, in a similar study, increases in task workload were associated with beta activity across midline electrodes [27]. Furthermore, beta has been associated with arousal [28], attentional gating [29], increased memory for advertising [30], and individual preference and commercial success [31], all consequences of increased immersion. Whilst these accounts support the notion that beta-band oscillations are related to an individual’s engagement with a task, these findings are relatively limited within consumer contexts and even more so within audio consumption research.

Attitude is arguably the most commonly cited and well understood metric within consumer contexts. Positive attitudes are reported to be linked to a myriad of desirable brand outcomes. Specifically, positive attitudes towards branding information and advertising contexts are said to increase all aspects of the purchase funnel, including brand awareness, ad recall, consideration, and purchase intent [32]. Additionally, positive attitudes are associated with brand loyalty, willingness to pay, and brand choice, amongst other factors [29]. Given the importance of attitude in driving changes in brand perception and, thus, consumer behaviour, it is important for brands to understand whether the audio format they select will influence consumer/brand perceptions.

According to Davidson, motivational, approach, and withdrawal processes are related to alpha activity across left and right prefrontal cortexes, respectively [33]. Within consumer contexts, such findings have been reiterated, with left frontal alpha activation reported to be related to affirmative purchase intent, higher willingness to pay, and an increased perceived need for a product [34]. Additionally, left anterior activation has also been associated with higher brand recall [35] and increased brand loyalty [36]. Conversely, higher levels of right anterior activation have been associated with lower brand recall [35], lower purchase intent, reduced willingness to pay, and a reduction in the perceived need for a product [34]. Of most importance to the present research, however, are the reports that media, specifically television content, elicits higher left frontal alpha activation when processing positive/pleasant content, and higher right frontal alpha activation in the presence of negative/unpleasant content [37,38,39,40]. Despite these findings relating to audiovisual stimuli, there is reason to suggest that the diagnosis of long form content and advertising across other mediums be accomplished via the use of neuroscientific methodologies.

All advertising is aimed at driving memories. Mental availability, a concept entirely dependent on memory formation, describes the propensity of a brand to be thought of or noticed in a buying situation [41]. It is generally accepted that as brands become more established in the minds of consumers, neural networks become stronger, increasing the likelihood that they are eventually selected. Therefore, understanding the neural underpinnings of memory is incredibly important for brands. Existing literature has demonstrated that the contexts within which ads are placed plays a pivotal role in the formation of brand-related memories [42]. These pieces of research highlight the role of memory in driving behaviour and, thus, the requirement of brands to understand their neural underpinnings. The most commonly cited model used to understand the neural components of memory is that provided by Tulving et al., which suggests a hemispheric encoding/retrieval asymmetry (HERA). Specifically, it is hypothesised that the left prefrontal cortex is associated with the encoding of episodic memories, whilst the right prefrontal cortex is associated with the retrieval of episodic memories [43]. Subsequent literature has expanded on these findings and implicated theta band oscillations as relevant to the encoding and retrieval of information [44,45,46,47,48,49]. Within consumer contexts, Vecchiato et al. revealed that anterior theta activity was related to memory components of political speeches [50]. The findings suggested that this activity may have been related to memorisation processes, and that the results of the study could likely be used to predict the victory of the election with a precision of 68.8%. To further substantiate these results, Silberstein and Nield confirmed that long term memory encoding was associated with left prefrontal sites whilst participants viewed commercials for common store bought items [51]. Similarly, Shestyuk et al. reported that increases in anterior theta activity, associated with memory, were positively correlated with television viewership and subsequent Twitter activity related to the television content [52]. Finally, Kong et al. revealed that the effectiveness of television commercials could be measured via the assessment of anterior theta oscillations [53]. Together, these findings highlight the relevance of neural measures in understanding memory processes and the predictability of outcomes within consumer contexts.

In addition to the above metrics, the media industry has shown considerable interest in understanding attention towards advertising. The development of mental availability and, thus, the propagation of consumers throughout the purchase funnel is reliant on attention. In other words, without attention, brands cannot exist within the minds of consumers. However, consumers are thought to be exposed to thousands of ads daily. The implication of this overwhelming exposure is that consumers are becoming much more capable of detecting and, thus, ignoring ads (i.e., ad aversion). This understanding has prompted the formation of numerous global attention vendors. Attention vendors allow brands to understand the level of attention that their advertising garners via the use of eye-tracking. Despite the value that these vendors provide, the inability to measure visual attention within the audio space is problematic. It is well established that the benefit of audio is obviously that, regardless of whether it is actively listened to or passively consumed, it has the capacity to change behaviour [18]. Neuroscience provides a valuable resource to the audio industry because it allows for a standardised metric by which to measure the attention given towards all forms of advertising.

A large body of research has revealed the consistent nature by which attention can be measured using neuroimaging techniques [54,55,56,57], particularly within the visual domain. Specifically, a large portion of this research implies that decreases in, or the suppression of, alpha activity is positively correlated with the strength of attention towards a stimulus [58,59,60]. Moreover, additional papers have exposed links between alpha activity, neural efficiency, increases task performance [61], and selective processing [62,63]. Of interest to the current paper is the translational nature of these findings across audio domains. To date, very little research has looked to investigate the commercial benefits of utilising neuroscience methods to understand attention towards audio formats. That which has been conducted, albeit outside of consumer contexts, alludes to alpha playing an influential role in increased effort and/or processing [37,64,65,66,67,68,69].

Of final interest to the current paper is how the physiological state of the consumer drives brand perceptions and consumer behaviour. Existing literature demonstrates clear links between media content and the physiological state of the consumer [70]. Higher levels of arousal have not only been shown to initially inhibit deep processing related to advertising and, as a result, to increase their persuasiveness [70], but it has also been shown that higher states of arousal are positively correlated with higher long term recall [70]. Despite the relevance of physiological markers, it is acknowledged that this approach does not allow for a complete understanding of emotional valence [71]. Specifically, arousal provides a measure of how intense a particular emotion is, but it does not provide insight into the valence of the emotion (i.e., whether the emotion it positive or negative) [72]. Whilst the authors acknowledge the importance of emotional valence in assessing consumer behaviour, it was not included during the analysis. As a result, emotional valence falls outside the scope of the current research.

### Radio, Podcast, and Music Consumption, and Processing

**Figure 1:**
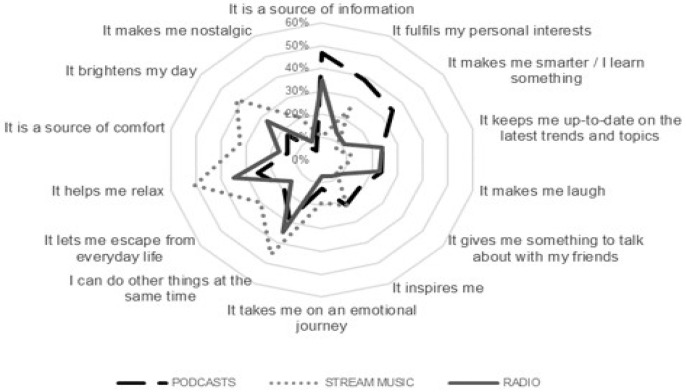
Self-reported use cases of radio, podcasts, and music streaming. This graph was produced by Australian Radio Network in 2019, summarising the results of 2027 individuals aged between 18 and 54 outlining their consumption habits of radio, podcasts, and music streaming.

It is easy to understand the difference between radio, podcasts, and music streaming. Where both radio and podcasts are fundamentally based on the spoken word, music streaming is a combination of spoken words and music. The strengths of radio and podcasts lie in their diversity. Radio messages are fleeting and conditioned by programming, temporal conditions, and subject limitations, and are mostly broadcast live. By contrast, podcasts are recorded files with more specific content that listeners can freely choose. Additionally, there are no limitations on the topics, and, given that they are recorded files, consumers can listen when and where they like, with the possibility to later subscribe to the content. These differences result in diverse motivations to listen. Australian Radio Network (ARN) conducted a survey of over 2000 individuals and reported that podcasts were primarily utilised as a means of fulfilling personal interests and keeping informed, whilst music streaming was typically engaged to elicit emotional responses [73]. Interestingly, radio was reported to have been utilised for personal interests and emotional reasons, as shown in Figure 1.

The current paper aims to extend the findings of this previous research and support the notion that each medium presents a new opportunity for marketers and advertisers from a neurological perspective. Whilst these findings are not currently available in the existing literature, there are numerous studies that investigate audio consumption habits. It is generally thought that people listen to music for enjoyment and pleasure [74], to pass the time and relax [75], to make social interactions [76,77], and for educational purposes [78,79]. These reasons, whilst similar, are slightly different to those given for podcast consumption. According to Edison Research [80,81], people listen to podcasts mainly for entertainment, educational reasons, and relaxation. The most popular topics are music and news/information. McClung and Johnson concluded that the reasons for listening to podcasts are entertainment, time-shifting, library building, and social aspects [82]. Podcasts have been shown to be an effective learning tool [83], which is one of the main reasons millennials consume them [84]. And, with regards to radio, in line with the study conducted by ARN [66], people listen to have company and feel well [5], derive psychological benefits, and remain informed. Li et al. have shown that audio formats complement one another, with users typically listening to multiple formats [85].

Considering this data, the research question of this paper is whether the diverse features of individual audio formats can lead to different processing. When assessing the success of each medium, engagement, attitude, arousal, attention, and memory (encoding and retrieval) are commonly utilised metrics in neuroscience-based advertising.

Some research has found that music can draw attention to an external focus, improve enjoyment, and induce positive memories [86,87,88,89]. Other studies conducted through recording EEG signals have also found that music stimulates positive emotional responses [86,90,91]. However, when it comes to enjoying radio news stories, the background music might not always have a positive effect. Carpentier found that, in most cases, the enjoyment of radio news was higher with no background music than with music [92]. However, no studies have compared these formats’ engagement, as far as we know. Consequently, we formulate the first research question:

- Q1. Which audio format will elicit the highest level of engagement?

The type of audio content might also have a differential effect on the attitude of the individuals. With the emergence of new technologies, a recent study found that young adults have a more positive attitude towards MP3 music, followed by AM/FM radio, than towards online streaming radio [93]. A more recent piece of research confirmed that young people showed a more positive attitude towards music streaming services [94], followed by offline broadcast radio, than towards broadcast radio apps. However, research comparing the three types of audio content is scarce. Due to the lack of previous evidence, the following research question is formulated:

- Q2. Which audio format will elicit the most positive attitude?

In the current study, we are also interested in the different effects of the type of format on attention and memory. First, with regards to memory and radio, previous research showed that people remember more information from advertisements when products are presented on radio than in printed media [95]. A more recent experiment comparing music and radio showed that drivers recalled more information when listening to music than when listening to a radio documentary [96]. Similarly, Allan found that popular music in radio ads enhances memory [97]. As a result of this literature, we postulate the following question:

- Q3. Which of the three audio formats will be more aligned with memory processing?

As for attention, it has been found that podcasts require enhanced concentration, while music is more relaxing [98]. Accordingly, Cabañero et al. observed that listening to a podcast was one of the tasks with a higher mental effort associated [99]. An EEG-based cognitive load analysis found that audio consumption requires a higher cognitive load than other activities, including making a call or reading a text message. Likewise, Super et al. found that listening to recorded speech avoids attention drifting (i.e., loss of attention) while driving, in an experiment with seven adults [100]. These findings were further supported by a more recent experiment which demonstrated that participants showed more attention when listening to a radio talk programme than when listening to music [101]. Due to the diverse results found in the few studies conducted about these formats, we formulate the following research question:

- Q4. What kind of format will achieve more attention and recall?

Arousal is defined as any change in consciousness as a result of a specific stimulus, which reflects a change in the body’s preparedness to act [102]. To date, arousal has been utilised within commercial settings to assess in-store atmospheres [103,104], effects of fast-paced and slow-paced music [105], television consumption [106], and advertising effectiveness [107]. Music has been shown to increase physiological arousal regardless of volume level [108]. However, what is of particular interest to the present research is the notion that measures of arousal offer insight into the intensity of the particular emotion experienced by the user towards radio and television [109]. According to these authors, radio and television evoke similar levels of intensity, but television elicited a slightly higher negative emotion.

Focusing on the comparison among different audio content formats, Bigliassi et al. studied the cerebral responses to music and podcasts during physical activity [110]. The results showed that music elicited more positive affective responses, increased arousal and enjoyment, regulated beta waves, and led to more dissociative thoughts. On the contrary, podcasts did not affect perceptual and affective responses. Similar studies have also found that music, in comparison to audiobooks or podcasts, increased dissociative thoughts during exercise and induced more positive emotional responses [88,111].

To add to these findings are those pertaining specifically to music, which have repeatedly reported changes in skin conductance (SC), heart rate (HR), and respiration (RSP). There is a large body of literature which has demonstrated that audio, specifically music, elicits significant changes in arousal [112,113,114,115]. For instance, Trappe reported that classical music was associated with decreases in heart rate and blood pressure [116]. Additionally, Armon et al. demonstrated that physiological changes occur as a result of having listened to rock music and classical music [117], whilst Edworthy and Waring reported similar findings, including that the pace and loudness of the audio resulted in changes to levels of arousal [118]. Together, these findings suggest that physiological states, in addition to neurological states, are susceptible to changes in audio format. That being said, no research, to our knowledge, has tested the differences in arousal towards the three most popular audio formats—radio, podcasts, and music streaming. This gap in the literature resulted in our fifth and final question:

- Q5. Which of the audio formats will result in the highest levels of arousal?

In sum, the main goal of the paper is to understand how radio, podcasts, and music streaming differ from a neurological and physiological point of view. If subtle differences exist in how each audio format is processed, then it is feasible that different opportunities to not only connect with consumers but influence behaviour exist between audio formats. Considering the scarce previous literature, we have formulated five research questions instead of hypotheses.

## 2. Materials and Methods

### 2.1. Participants

Sixty participants (30 male) were recruited for the current research via an external recruiter located in Sydney, Australia. Participants were aged between 25 and 54 (*M* = 36.45; *SD* = 9.07) and matched for age and gender. This age range was selected because they are the most sought-after audience amongst Australian radio advertisers. All participants volunteered and gave written, informed consent. Participants were right-handed, had normal or corrected-to-normal vision, were free of central nervous system affecting medications or substances (including alcohol, caffeine, and nicotine), and had no history of neuropathology. Participants were also required to regularly listen to audio, and not consider themselves a ‘rejector’ of audio. Participants were financially reimbursed approximately $80 for their time and travel.

### 2.2. Audio Stimuli

For the current experiment, three audio formats were selected. Specifically, each participant was exposed to two of the following three audio formats: radio, podcasts, and music streaming. To maintain consistency in the audio that was presented to participants, each piece of audio was exported as a .wav format, resampled at a rate of 44.1 kHz, had a bitrate of 320 kbps, and played at the same volume. Each audio format was designed to replicate a real-life listening experience.

#### 2.2.1. Radio

**Figure 2:**
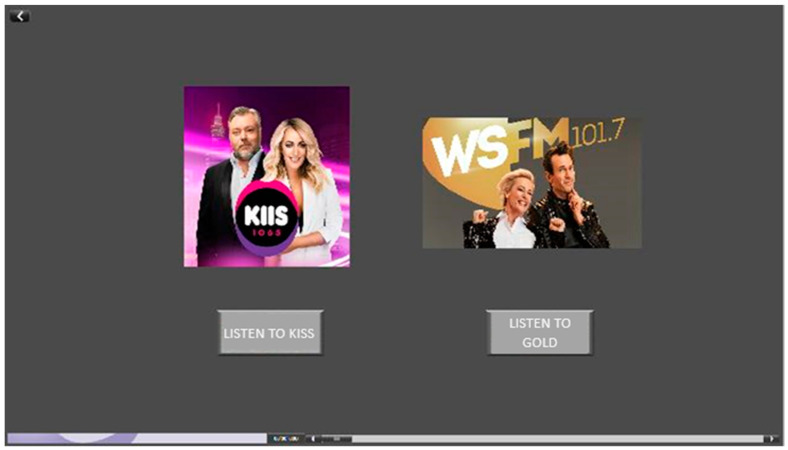
Example of radio selection screen presented to participants.

For those who were tasked with listening to radio, two radio segments were taken from two well-known Sydney-based radio stations, KIIS1065 and WSFM1017. Participants were given the opportunity to select one of the two stations to listen to (see Figure 2). Australian Radio Network provided the researchers with approximately 12 min of previously aired radio, split into two files. Advertisements belonging to ten well-known brands were then placed on either side of the two radio segments. For the purpose of the current study, the ads were of no interest to the researchers. For participants, the experience sounded as though they were listening to a live piece of radio being played on the air.

#### 2.2.2. Podcasts

**Figure 3:**
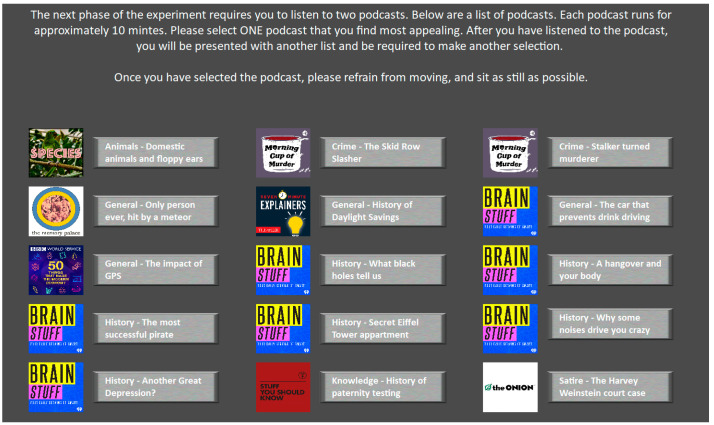
Example of the one of the selection screens presented to participants prior to the podcast segment.

For those who were tasked with listening to podcasts, a selection screen which listed 15 podcasts varying in genre (i.e., crime, animals, comedy/satire, interviews, general knowledge, and history) was presented. Each podcast ran for approximately eight minutes. Participants were instructed to select one podcast from a list of 15 (see Figure 3 for an example of the selection screen). After listening to the entire podcast, participants were presented with another screen where they were instructed to select a second podcast from another list of 15, again varying in genre. Each podcast phase of the experiment contained advertisements (pre-roll, mid-roll, and post-roll). However, their analysis falls outside of the scope of the current paper. The experience for participants was intended to replicate one typically experienced whilst listening to a podcast on iHeartRadio.

#### 2.2.3. Music Streaming

**Figure 4:**
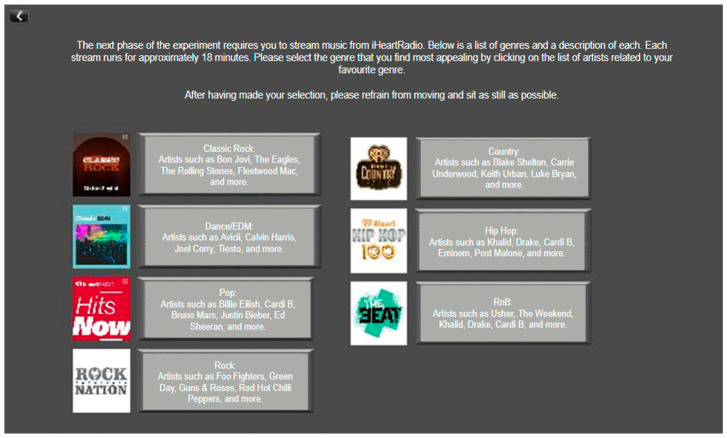
The selection screen presented to participants prior to the music streaming segment beginning.

For those who were tasked with listening to music streaming, a list of different music genres was presented. Participants were asked to select a genre of music from a selection screen similar to that experienced during the podcast segment of the study. Genres included classic rock, hard rock, EDM (i.e., Dance Music), pop, hip hop, and RnB. Each genre was presented with a brief description of music and a list of the artists belonging to each of the genres (see Figure 4 for the selection panel presented to participants). Once again, participants were exposed to advertisements throughout the listening experience, but the results are not reported within the current research piece. Overall, the music streaming segment was typical of an iHeartRadio music listening experience.

### 2.3. Lab Experiments

Following the completion of a recruitment survey provided by an external recruiter, eligible participants were asked to attend the offices of ARN, located in North Sydney. Participants were required to complete a single session, during which implicit measures were collected using EEG. Participants were assigned to groups based on their attendance date. The first 20 people to participate were assigned to group one, whilst the next 20 were assigned to group two, and the final 20 were assigned to group three. Each of the three groups was matched for age and gender, and was presented with a different combination of audio formats (see Table 1 and Table 2 for group allocation and task allocation). Upon entering the lab, participants were seated comfortably in front of a 32-inch LED television (resolution of 1020 × 1980 pixels). Participants were connected to a Mind Media NeXus-32 EEG system (Mind Media, BioTrace, Steenwijk, The Netherlands) and a pair of Sennheiser noise-cancelling headphones. After being fully set up, brain potential changes were measured using 21 cranial electrodes as well as two external reference electrodes supraocular (above the eyes) and infraocular (below the eyes). Electroencephalography changes were recorded using BioTrace+ (Version V2018A1; computer software from Mind Media BV) at a sampling rate of 512 samples per second. To visually present the individually prepared stimuli, we also utilised the BioTrace+ software. Although participants had been provided with documentation outlining the study, the specifics of the study were explained to participants while the sensors were attached and all cables were connected.

Once set up, participants were presented with the first of two audio segments depending on their group allocation. For group allocation and tasks, see Table 1 and Table 2, respectively. Participants were exposed to the first audio segment and, following a short break, were then presented with the second segment.

### 2.4. Variables

The dependent variables of this study are engagement, attitude, memory, attention, and emotional response (arousal and valence). We have measured these variables with different research tools, mainly EEG. Within consumer contexts, EEG is one of the most prevalent assessment tools utilised to understand the neural correlates of consumer behaviour. EEG has been used to assess marketing stimuli, including, but not limited to, media involvement [119], changes in brand attitude [120], the processing of TV commercials [121], and the prediction of memory for components of TV [35,122].

### 2.5. Signal Analysis & Data Analysis

All participant data were collected via the use of the MindMedia NeXus-32 EEG system. Data analyses focused on the differing audio segments (i.e., radio, podcasts, and streaming). After having removed eye movement and blink artefacts from the raw EEG data, the time series data associated with the audio segments were analysed for all 60 participants. Mean power was extracted for each frequency. Baseline power measures were taken from 3000 ms block, which had been previous presented universally to all participants. Following a Fast Fourier transformation, frequency bands for Alpha (8–13 Hz), Beta (13–25 Hz), and Theta (4–8 Hz) were extracted and normalised means were computed for each audio segment, reducing variance in the data to values ranging between 0 and 1. Typically, EEG data is collected in microvolts. However, for the purpose of this study, all data was normalised to allow for easier comparisons across each of the formats.

Each metric, as mentioned above, required the averaging of numerous electrode sites. The previous literature has demonstrated that averaging of electrode sites produces a global region-of-interest within each frequency band that is more robust than deriving and examining power at individual sites [53,123,124]. Data were initially collected at a sampling rate of 512 samples per second.

To assess attitudinal effects (i.e., frontocentral asymmetry), natural log alpha power values were averaged across right (sites F4 and F8) and left (sites F3 and F7) sites, separately (see Coan & Allen, for review) [125]. Subsequently, attitude was calculated using the following formula, in line with the pre-existing literature.

$$Attitude=\frac{\log\left(F3+F7\right)-log(F4+F8)}{\log\left(F3+F7\right)+log(F4+F8)}$$

With regards to attention, averaged alpha activity across frontal and parietal sites F3, F4, F7, F8, Fz, C3, C4, and Cz were of interest and, thus, calculated. This approach was in line with existing approaches [56,67,126]. Specifically, alpha band activity was isolated and calculated for each of the audio formats and each of the participants, separately, before being aggregated for analysis.

Memory related brain responses were derived in accordance with the widely adopted and referenced hemispheric encoding/retrieval asymmetry (HERA) model [43]. According to the HERA model, left prefrontal cortex (PFC) is said to be more involved than right PFC in episodic memory encoding, whilst right PFC is more involved than left PFC in episodic retrieval. Memory encoding and memory retrieval were processed via analyses of averaged theta activity across left frontal sites F3 and F7 and right frontal sites F4 and F8, respectively [127,128].

Given the strong links between beta activity and consumer engagement, beta activity was isolated and assessed during each audio format. The average beta activity across frontal sites was assessed. Specifically, engagement/alertness was assessed via the averaging of beta signals across electrode sites FP1, FP2, F3, F7, F4, F8, and Fz, in accordance with the previous literature [52].

In addition to metrics derived from EEG, physiological responses (heart rate, skin conductance, and respiration) were collected via a MindMedia NeXus-32. All physiological data was collected at a rate of 32 samples per second. BVP signals were collected using a sensor placed on the left index finger of participants. BVP was used to determine heart rate (HR) via peak-to-peak detection [129]. Heart rate sets that were predominantly below normal human rates (<40 beats per minute) were manually removed from the data set. Skin conductance (SC) was collected via two electrodes placed on the participant’s left middle and ring finger. Lastly, respiratory rates were collected via a respiratory belt placed around the participant’s chests, were analysed on a per hour scale, and were subsequently converted to per minute counts for ease of analysis.

Given the length of the stimuli being tested and the varied lengths of each, each audio piece was shortened to an arbitrary 30 data points in order to implement a level of consistency. Of all audio formats, the fewest data points were collected for radio. As a result, datapoints were randomly removed from the remaining audio groups until each contained an equal number of samples. The final analysis included a total of 10,500 data points for each audio format and 6300 data points for each metric.

## 3. Results

### 3.1. EEG

**Figure 5:**
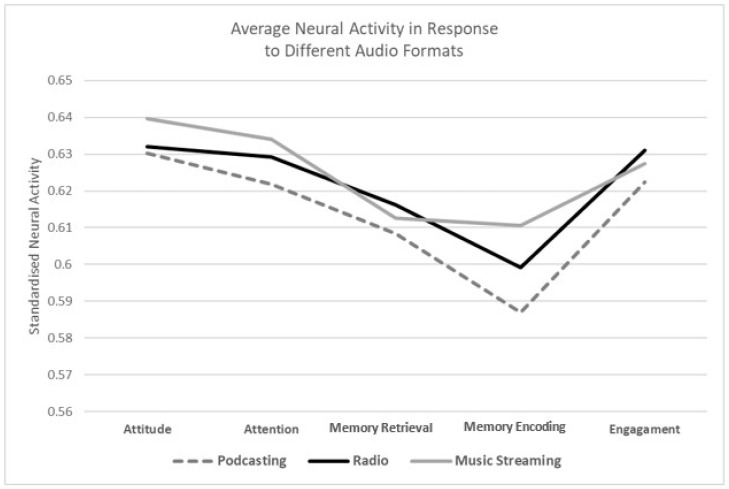
Average responses across each of the audio formats for each of the different metrics tested.

To begin, a two-way ANOVA was conducted to investigate main effects of each audio type (see Figure 5 for visual representation of findings). The results revealed a significant main effect of both audio type (*F*(2, 34185) = 9.86, *p* < 0.001, η^2^ = 0.001) and metric (*F*(4, 34185) = 38.61, *p* < 0.001, η^2^ = 0.005). Pairwise, comparisons were then undertaken and revealed that radio (*M* = 0.62, *SD* = 0.17) and music streaming (*M* = 0.62, *SD* = 0.20) elicited significantly greater activity, regardless of metric, than that of podcasts (*M* = 0.61, *SD* = 0.18). Further analyses were conducted to assess interaction effects across audio type and metric. The results revealed a significant audio type by metric effect (*F*(8, 31485) = 1.27, *p* < 0.001, η^2^ < 0.001).

Randomisation tests were then utilised to assess and further validate the individual differences between the three audio formats and the metrics tested. These kinds of tests are considered a more robust and accurate alternative to traditional *t*-tests [130]. For each randomisation test, 2000 permutations were conducted. Initial assessment of the different metrics revealed that listener engagement, despite being highest for radio, did not significantly differ between the three audio formats. Subsequent analyses investigated attitudes and, as expected, demonstrated that attitudes towards music streaming (*M* = 0.64, *SD* = 0.19) were more positive (attitude) than either podcasts (*M =* 0.63, *SD* = 0.18) or radio (*M =* 0.63, *SD* = 0.16; *t*(4198) = −1.70, *p* = 0.045, one-tailed, η^2^ < 0.001). These results address RQ1 and RQ2, regarding the effect of the audio format on listeners’ engagement and attitude. They show that, despite the visual differences in the graph, there is no significant difference in the level of engagement elicited by the different audio formats. On the contrary, music streaming elicited more positive attitudes than radio and podcasts.

Retrieval processes revealed no significant differences. However, resource allocation to encoding the different audio pieces saw music streaming (*M =* 0.61, *SD* = 0.20) elicit significantly more activity than radio (*M =* 0.60, *SD* = 0.19; *t*(4198) = −1.91, *p* = 0.056, two-tailed, η^2^ =< 0.001) and significantly more activity than podcasts (*M =* 0.61, *SD* = 0.20; *t*(4198) = −3.93, *p* < 0.001, two-tailed, η^2^ = 0.004). Additionally, radio was seen to elicit significantly more memory encoding processes than podcasts (*M =* 0.59, *SD* = 0.19; *t*(4198) = −2.11, *p* = 0.035, two-tailed, η^2^ = 0.001). Thus, we can answer RQ3 by concluding that music streaming is more aligned with memory processing than the other audio formats.

The final aspect of EEG analyses, seen to focus on attention, saw resources allocated to attending to the stimuli greatest during music streaming (*M =* 0.63, *SD* = 0.20) than they were during radio (*M =* 0.63, *SD* = 0.17) or podcasts (*M =* 0.62, *SD* = 0.18; *t*(4198) = −2.10, *p* = 0.036, two-tailed, η^2^ < 0.01). These results answer RQ4 by showing that music streaming achieved more attention than radio and podcasts.

### 3.2. Physiology

Physiological measures including BVP, respiration, and skin conductance were collected and analysed in a similar manner to the EEG data. Each audio listening segment was averaged to 30 data points for consistency and analysis was conducted across all data points. Initial analysis of BVP revealed significant effects across the different music types (*F*(2, 5037) = 654.90, *p* < 0.001, η^2^ = 0.206). Multiple comparisons revealed that music streaming (*M* = 10.99, *SD* = 45.9) elicited significantly higher BVP readings than either podcasts (*M* = −21.28, *SD* = 16.66) or radio (*M* = −20.54, *SD* = 15.05; *p* < 0.001, η^2^ = 0.206). To further assess the effects of BVP, the data was converted to heart rate (HR; beats per minute) and an ANOVA was used to compare the three different audio types. The results revealed a significant effect of audio type on HR (*F(*2, 3687) = 34.21, *p* < 0.001, η^2^ = 0.018). Pairwise, comparisons using Fisher’s least significant difference (LSD) revealed that music streaming elicited a significantly greater HR (*M* = 54.12, *SD* = 7.23) than either radio (*M* = 51.97, *SD* = 8.31; *p* < 0.001) or podcasts (*M* = 53.10, *SD* = 5.95; *p* < 0.01).

With regards to skin conductance (SC), a one-way ANOVA revealed a significant difference between the three mediums (*F*(2, 5037) = 632.49, *p* < 0.001, η^2^ = 0.201). Radio was seen to elicit the highest SC response (*M* = 9.67, *SD* = 42.92), followed by podcasts (*M* = −1.84, *SD* = 2.07), and then music streaming (*M* = −21.59, *SD* = 16.20; *p* < 0.001).

Respiration, the last physiological measure recorded, revealed that music streaming (*M* = 624.61, *SD* = 51.53) elicited significantly higher respiration peaks than either radio (*M* = 619.48, *SD* = 53.18) or podcasts (*M* = 617.26, *SD* = 61.55; *F*(2, 5037) = 7.71, *p* < 0.001, η^2^ = 0.003). To further establish the effects of audio type on respiration, the values were converted to breaths per minute. A one-way ANOVA was utilised to investigate the effects of audio type on respiration rate. A significant effect of audio type on respiration was seen (*F*(2, 5037) = 7.71, *p* < 0.001, η^2^ = 0.003). Pairwise, comparisons using LSD revealed that music streaming (*M* = 624.61, *SD* = 51.53) elicited significantly higher respiration rates when compared to both podcasts (*M* = 617.26, *SD* = 61.55; *p* < 0.01) and radio (*M* = 619.45, *SD* = 53.18; *p* = 0.008). These results answer RQ5 by demonstrating that music streaming elicited the highest rates of physiological arousal across two of the three measures of arousal utilised.

## 4. Discussion

The goal of this study was to analyse whether the listeners’ engagement, attitude, attention, and memory changed when exposed to three different audio formats (radio, podcasts, and music streaming). The existing research within the field of audio has focussed heavily on music, largely due to its ability to encourage emotional responses [52]. However, podcasts and radio have seen less interest from an academic perspective despite their apparent differences from music streaming. Specifically, radio and podcasts are, at a conscious level, thought to evoke different affective and motivational responses in the listener. The current research demonstrates that neurophysiological tools are sensitive enough to determine differences in how each audio format is processed. Overall, results showed that music streaming induces a greater psychological response than the other audio content. The findings are summarized and discussed below.

This study adds to the existing literature, which has predominantly derived its findings from traditional, survey-based approaches, and demonstrates that different audio formats result in different neurological states. Advertising aims to drive changes to consumer behaviour, with the final outcome resulting in a purchase. And, whilst this is obvious, brands are largely unaware that it is the brain that is responsible for these changes. Often, this relationship between the brain and outcomes is neglected, and without an understanding of how the organ responsible for behaviour (i.e., the brain) functions, it is inherently difficult to initiate change. In other words, changing behaviour is reliant on changes at a neuronal level and, for that reason, it is imperative that brands understand how the brain responds.

Firstly, regarding the level of engagement elicited by the audio formats analysed, despite the visual differences in the graphs, the results indicated that there were no significant differences between music streaming, podcasts, or radio. Interestingly, this suggests that all audio formats possess the capacity for high engagement. However, despite our study showing that the level of engagement did not significantly differ between audio formats, radio appeared to elicit the strongest levels of engagement. This trend in the data is unsurprising, given the way that radio is consumed. Radio cements bonds between the listener and the talent. According to Peacock, Purvis, and Hazlett, radio drives higher levels of engagement than television [109]. Further to these findings, it is posited that the use of persistent talent with whom consumers bond within radio is one of the leading factors contributing to higher levels of engagement. Moreover, other studies have confirmed that celebrities do, in fact, drive higher levels of engagement [131,132,133], and this high engagement results in more positive brand associations [133]. The current research demonstrates that, whilst not significantly, radio elicits higher levels of engagement than other audio mediums and, thus, presents a unique opportunity for brands to elicit stronger consumer/brand relationships.

Secondly, our results show that music elicits more positive attitudes in comparison to radio and podcast. These findings are consistent with previous research showing that young people have a more positive attitude towards music or music streaming services than towards other audio formats [93,94]. Attitude is inherently valuable for brands, given its link with brand loyalty, willingness to pay, and brand choice [29]. Considerable research has demonstrated that positive environments encourage more positive emotions, which, in turn, increase the likelihood of positive brand associations [134,135,136]. In a society where consumers find the majority of advertising intrusive and negative [137], brands must adopt tactics that mitigate negative interactions. The findings of the current paper highlight the positive nature of music streaming and the opportunity for brands to increase positive brand associations, simply by selecting different channels.

Thirdly, in line with the previous literature, music streaming saw the highest levels of memory encoding than all audio formats [96,97]. Interestingly, podcasts, a medium that is generally curated to educate (and promote the storage of new information), saw the lowest levels of memory encoding. Whilst these findings seem counterintuitive, popular music is typically listened to repeatedly and is capable of developing strong neural networks [126]. Neural networks are strengthened via repeated exposure. Given how frequently consumers of audio listen to their favourite songs, it is unsurprising that music streaming elicited the highest levels of memory encoding. It is possible that the metric utilised within the current study to determine the level of memory encoding provided insight into memory more generally, instead of the specific memory related processes that occurred as a result of listening to the audio. In other words, when listening to songs that were already well-established in the minds of consumer, memory was naturally higher than during a task that was designed to induce memory encoding. A second plausible explanation for the current findings related to lower levels of memory encoding during podcasts is that the content was not appealing to the participants. Although participants were able to choose from 30 podcasts, podcasts are extremely personal, and the variety typically offered within podcast applications is significantly greater. Therefore, it is possible that consumers found the podcasts of little interest and chose not to commit any of the content to memory.

Fourthly, music streaming elicited the highest levels of attention when compared to either podcasts or radio. Attention has become such a highly sought after commodity, given consumers’ daily bombardment by ads. Advertising avoidance has become such an issue that brands have begun to optimise towards attention [138]. The media landscape has changed considerably, and brands acknowledge the importance in capturing consumer attention in order to drive outcomes. Commercial research has shown that the environments within which ads are placed set the boundaries of attention. In other words, certain environments lend themselves to either higher or lower attention durations, and advertising within these mediums is constrained by these limits [139]. The current research demonstrates that the attention commanded by music streaming and radio is higher and, thus, posits that consumers are better positioned to attend to messages within channels.

Finally, and following previous findings in the literature, our results show that music streaming elicited the highest rates of physiological arousal compared to radio and podcasts. This is consistent with the study by Bigliassi et al. which found that music induced high levels of physiological arousal [110]. Within consumer contexts, higher levels of arousal have been associated with the inability to deeply process advertising related material, resulting in increased persuasiveness. Furthermore, high arousal is reported to be positively correlated with greater long-term recall [70]. With regards to advertising implications, these findings promote potential benefits surrounding the tactical use of music to enhance branded messaging. As has already been mentioned, consumers have become increasingly adept at avoiding advertising content, and it is possible that the strategic implementation of music within advertising may promote more receptive audiences.

The current study demonstrated that radio, podcasts, and music streams present different consumer experiences and, thus, unique marketing opportunities. Overall, and from a neurological perspective, the study is the first to show that listeners’ perception and processing differ if they are exposed to different audio formats (i.e., radio, podcasts, or music streaming). The results show that whilst radio elicits the highest levels of engagement, music streaming is associated with more positive attitudes, memory processing, and attention than the other audio formats. Music streaming also elicited higher rates of physiological arousal in comparison to radio and podcast. Together, these findings emphasise the need for brands to consider how the channel in which they place their advertising influences the perception of their brand messaging.

## 5. Limitations & Future Research

Whilst the paper contributes significantly to the media industry’s understanding of different audio formats and provides actionable insights with regards to their adoption, there are limitations. Firstly, and most evident, is the content related to the listening experiences. Despite participants being provided with two radio stations, seven genres of music, and thirty podcasts, the music industry is significantly larger. It is possible that, despite participants having reported not to be an audio rejector, only a limited selection of the audio excerpts was of interest. Future research, where possible, should endeavor to provide participants with unvetted access to platforms that offer all of the audio channels offered within this study.

A second limitation of the present study is that which is common to all neuroscience research. Within lab settings, consumer behaviour may not reflect true behaviour in a real-world setting. Typically, when consumers consume audio, seldom are they sitting still, doing nothing but listening. It is possible that this affected our results. Future research should aim to collect data from participants whilst they consume audio in their typical environments or engaging in tasks during which they would typically consume audio.

A third limitation of the current research is the lack of consistency across different audio formats. Whilst the sample rate, bitrate, and volume were maintained across the different audio formats, other ‘physical’ features of the audio could have been controlled. Several studies have shown that variations in the physical attributes of audio stimuli can significantly influence the resulting EEG signal. Research investigating speech and song processing has demonstrated that the inclusion of speech information itself modulates the EEG response [140]. Furthermore, sung words are processed differently than spoken words, as evidenced by distinct brain region activation patterns [141]. The complexity of the melodic content can also influence the EEG signal, with studies revealing variations in hemispheric lateralisation based on melodic richness [141]. Additionally, familiarity with the audio material seems to play a role, as suggested by research on familiar versus unfamiliar songs [140]. Finally, the way pitch is used, either for linguistic prosody or musical melody, appears to differentially activate brain regions [142], potentially reflected in the EEG signal. Beyond these findings, other physical properties of sound, such as timbre, rhythm, and overall volume, are also likely to influence EEG responses. Future research should explore these possibilities to gain a more comprehensive understanding of how the brain processes diverse auditory stimuli.

A final limitation of the present study is the lack of emotional metrics. Emotion is known to be a strong driver of brand perceptions and a robust measure of consumer experience, making it important within advertising contexts. However, despite the inclusion of EEG within the current study being used to determine neural components related to audio consumption, data were not analysed in a way that allowed for an assessment of emotion. Recent research has supported the notion that EEG can be implemented to discern dichotomous emotional states [72]. Future research should address this shortcoming by employing an implicit emotional measure, such as EEG, which offers sensitivity to underlying affect.

## 6. Conclusions

In conclusion, this study shows the differential impact of audio formats on consumer engagement and offers a nuanced understanding of strategic brand positioning in the auditory marketing domain. Engagement levels across radio, podcasts, and music streaming did not exhibit significant variations, suggesting an intrinsic capacity within all auditory media to secure consumer engagement. Remarkably, radio was associated with the highest engagement levels, which may prompt a reevaluation of the medium’s role in contemporary advertising strategies, particularly in its capacity to catch audience attention.

Music streaming emerged as a superior format in fostering positive attitudes, increasing attention, enhancing memory encoding, and elevating physiological arousal compared to its counterparts. These attributes highlight the potent emotional and cognitive influence of music streaming, indicating its preeminence in capturing consumer interest and sustaining brand recognition amidst the pervasive challenge of advertisement avoidance.

Conversely, despite their educational design, podcasts elicited the lowest levels of memory encoding. This unexpected outcome suggests a potential incongruence between podcasting’s intended function as a medium for knowledge dissemination and the actual cognitive engagement it facilitates.

The implications of these findings are multifaceted for marketers. Radio, podcasts, and music streaming’s distinct experiential dimensions manifest unique opportunities for crafting marketing communications. Music streaming’s profound engagement positions it as an effective conduit for attention-centric branding efforts. Furthermore, radio’s enduring engagement underscores its value in auditory marketing campaigns. A diversified approach, harnessing the distinctive advantages offered by each audio format, could thus be pivotal in optimizing brand impact and recall in the consumer’s brain.

## Figures and Tables

**Table 1:** Group allocation and task order.

Group One	Radio	>	Podcasts
Group Two	Podcasts	>	Music Streaming
Group Three	Music Streaming	>	Radio

**Table 2:** Visual representation of how the audio listening task was structured.

Group	Task Breakdown					
Radio	Radio Listening	Ad Block	Radio Listening	Ad Block		
Podcasts	Ad Block	Podcast Listening	Ad Block	Podcast Listening	Ad Block	
Music Streaming	Music	Ad Block	Music	Ad Block	Music	Ad Block

## Data Availability

Data relating to this study can be obtained via a request made directly to the authors. The data are not publicly available due to the data being owned by a private entity (Australian Radio Network; ARN).
